# Medication Incidents Related to Automated Dose Dispensing in Community Pharmacies and Hospitals - A Reporting System Study

**DOI:** 10.1371/journal.pone.0101686

**Published:** 2014-07-24

**Authors:** Ka-Chun Cheung, Patricia M. L. A. van den Bemt, Marcel L. Bouvy, Michel Wensing, Peter A. G. M. De Smet

**Affiliations:** 1 Scientific Institute for Quality of Healthcare, Radboud University Medical Centre, Nijmegen, The Netherlands; 2 Royal Dutch Pharmacists Association (KNMP), Den Haag, The Netherlands; 3 Department of Hospital Pharmacy, Erasmus MC, Rotterdam, The Netherlands; 4 Department of Pharmacoepidemiology and Clinical Pharmacology, Utrecht Institute for Pharmaceutical Sciences (UIPS), Utrecht University, Utrecht, The Netherlands; 5 Department of Clinical Pharmacy, Radboud University Medical Centre, Nijmegen, The Netherlands; Bielefeld Evangelical Hospital, Germany

## Abstract

**Introduction:**

Automated dose dispensing (ADD) is being introduced in several countries and the use of this technology is expected to increase as a growing number of elderly people need to manage their medication at home. ADD aims to improve medication safety and treatment adherence, but it may introduce new safety issues. This descriptive study provides insight into the nature and consequences of medication incidents related to ADD, as reported by healthcare professionals in community pharmacies and hospitals.

**Methods:**

The medication incidents that were submitted to the Dutch Central Medication incidents Registration (CMR) reporting system were selected and characterized independently by two researchers.

**Main Outcome Measures:**

Person discovering the incident, phase of the medication process in which the incident occurred, immediate cause of the incident, nature of incident from the healthcare provider's perspective, nature of incident from the patient's perspective, and consequent harm to the patient caused by the incident.

**Results:**

From January 2012 to February 2013 the CMR received 15,113 incidents: 3,685 (24.4%) incidents from community pharmacies and 11,428 (75.6%) incidents from hospitals. Eventually 1 of 50 reported incidents (268/15,113 = 1.8%) were related to ADD; in community pharmacies more incidents (227/3,685 = 6.2%) were related to ADD than in hospitals (41/11,428 = 0.4%). The immediate cause of an incident was often a change in the patient's medicine regimen or relocation. Most reported incidents occurred in two phases: entering the prescription into the pharmacy information system and filling the ADD bag.

**Conclusion:**

A proportion of incidents was related to ADD and is reported regularly, especially by community pharmacies. In two phases, entering the prescription into the pharmacy information system and filling the ADD bag, most incidents occurred. A change in the patient's medicine regimen or relocation was the immediate causes of an incident.

## Introduction

Aging is strongly associated with polypharmacy. [1], [2] In The Netherlands four in five people over 75 years use five times more medicines compared to the general population. [2], [3] Home-dwelling elderly patients can have several problems when managing medicines, such as vision or cognitive impairments, which make it difficult to differentiate between medicine packages. [4] Patients need support tools to use their medicines appropriately. Automated dose dispensing (ADD), also known as multi dose dispensing, is an example of such a tool. ADD provides patients with robot-dispensed unit doses. All medicines intended for one dosing moment are gathered in disposable bags and labelled with patient data, medicine contents and the date and time for intake. [5]–[7] Specific dosage forms (e.g., suppositories, oral liquid formulations) cannot be dispensed with this system. [8] ADD is frequently used in hospitalised patients across the world and has been introduced in primary care for the home-dwelling elderly patients in a range of countries, such as Denmark, Finland, The Netherlands, Norway and Sweden. [2], [5], [9]–[11]

ADD has been introduced aiming to improve medication safety and treatment adherence, particularly in elderly patients with multiple medications. Additional advantages of ADD are a reduced workload for the pharmacy dispensing staff and nurses administering the medication, avoidance of old stockpiles of medication at home, and decreased medication costs. [12] Early studies have confirmed that automated medication dispensing systems minimize medication dispensing errors and save time for the pharmacy dispensing staff. [13]–[16] Low error rates between 0.07‰ and 0.10‰ of automated dose dispensing machines have been observed during a 6-months follow-up period. [17] Other studies focused on treatment adherence and medication knowledge of the patient. [7], [9], [11], [18] Kwint et al showed that ADD users have a substantially higher self-reported adherence compared to non-ADD users (91% versus 58%). [7]

In addition to these positive effects, ADD may also introduce new types of medication errors. [8], [17], [19], [20] Two studies have shown that patients using ADD are at increased risk of receiving inappropriate medicines like long-acting benzodiazepines, anticholinergic medicines, and three or more psychotropic medicines. [5], [19] Van den Bemt et al studied the administration of medications in nursing homes that used ADD. From 2,025 medication administrations they detected 428 (21.2%) medication errors; the most frequently occurring types were wrong administration technique (e.g. incorrect crushing of medication) (n = 312, 73%) and administering the medication at least one hour early or late (n = 77, 18%). [8] These studies focused on incidents occurring in the medication administration phase. Overall insight into medication incidents related to ADD across the full range of phases of the medication process (from prescribing to dispensing, storage and administration) is still missing.

In The Netherlands a nationwide reporting systems for medication incidents, Central Medication incidents Registration (CMR), is available. [21] Medication incidents associated with ADD are reported relatively often to the CMR. This triggered us to explore this subject in more detail. In this descriptive study, we aim to provide insight into the nature and consequences of medication incidents related to ADD, as reported by healthcare professionals in community pharmacies and hospitals.

## Methods

### Data source

In The Netherlands, 93 hospitals and 1997 community pharmacies operated in 2012. [2], [21], [22] For this study we collected medication incidents that were reported in community pharmacies or hospitals between January 2012 and February 2013. The retrospective collection and analysis of the incidents are exempt from medical ethical approval by Dutch Clinical Trial law as they do not compromise the integrity of patients. All data were handled anonymously according to the privacy legislation in The Netherlands. [21]

### Setting

In The Netherlands hospital pharmacies generally serve both hospital beds and beds in nursing homes. Especially for the beds in nursing homes the hospital pharmacies use ADD to support the nurses in the administration of medicines. For the home-dwelling elderly patients using multiple medications the community pharmacies often dispense their medication using ADD.

**Figure 1.Scheme:**
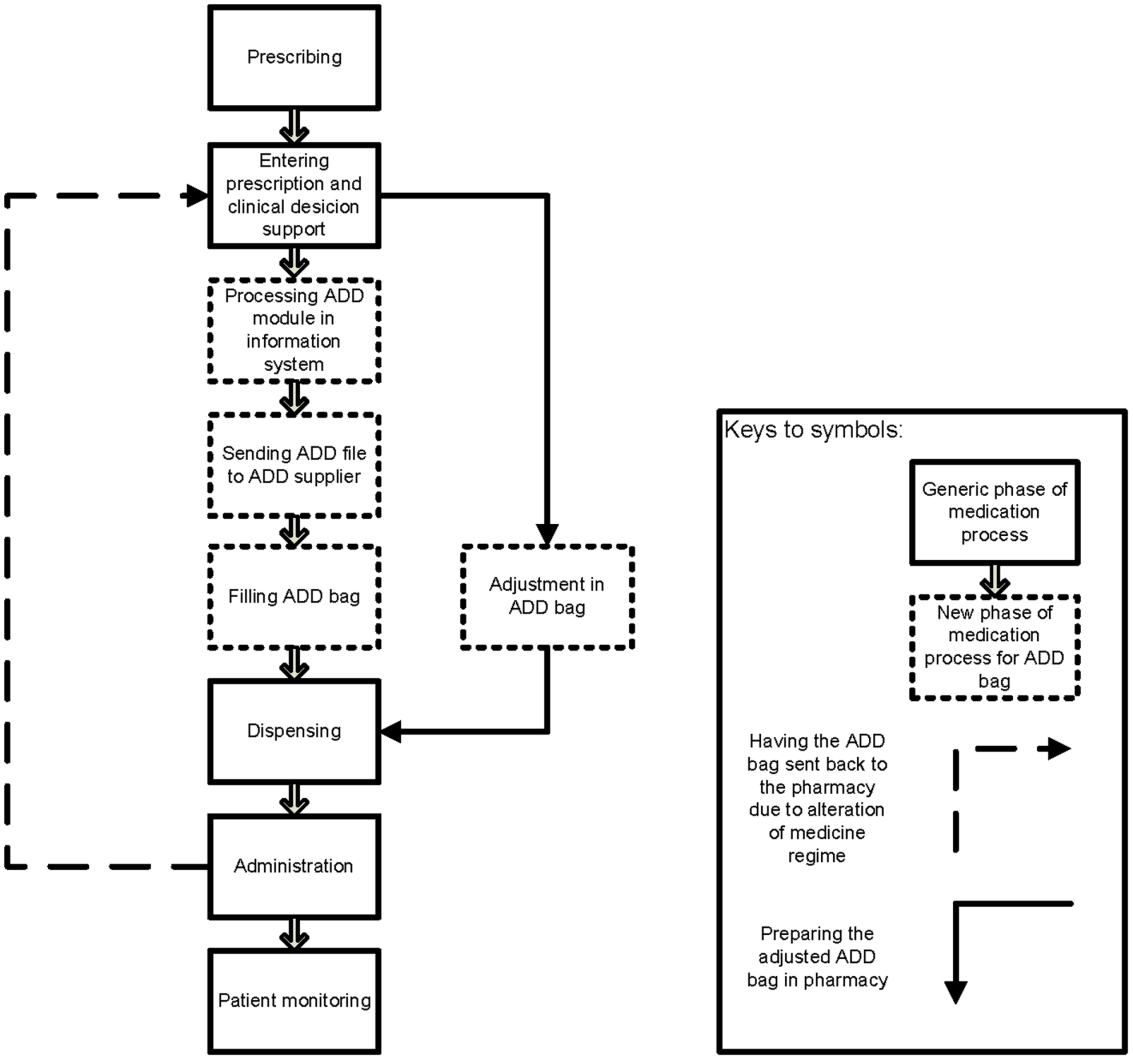
Scheme of the ADD process.

The ADD dispensing robots can be located in the hospital or community pharmacy itself, but especially community pharmacies tend to purchase this service from a pharmacy that is specialised in ADD (the latter will be referred to as the ADD supplier). The hospital pharmacist or community pharmacist will always remain responsible for entering the prescriptions into the pharmacy information system. Subsequently, the pharmacist transmits the ADD file electronically to the ADD supplier. Using this ADD file the ADD supplier fills the ADD bags. In the next step the hospital pharmacist dispenses the ADD bags to the nursing homes and the nurse administers the medicines to the patient according to an administration list. In the community pharmacies the ADD bags are dispensed directly to the patient and the community pharmacist provides counselling about the medicines and how to use the ADD bags. Some patients are supported in their administration of the medicines from the ADD bag by home care nurses. When an alteration (e.g. new or changed prescription) occurs the pharmacist has two options: the alteration can be effectuated in the next ADD supply or the pharmacist collects the ADD bag from the patient and manually changes the ADD content. See figure 1: Scheme of the ADD process.

### Identification of relevant incidents

The CMR team analyses all submitted medication incidents reports every week to identify potential alerts and other outputs like an item in a newsletter. [21] The CMR team uses a home-made software programme during analysis and links notes to the report without changing the original description of the medication incident. Since October 2011 the CMR team has marked all the incidents that are associated with ADD and these incidents were selected for this study. Some reports were excluded from this study because these reports had been marked incorrectly by the CMR team due to an unclear description of the incident and/or confusing terms. From the incidents obtained from community pharmacies one duplicate was removed and from the hospital reports five duplicates were removed.

### Analysis of incidents

For each incident, one researcher (KC) collected directly from the incident report the patient's characteristics of the incident: gender, and birth year of the patient. Gender and year of birth of the patient (see Appendix S1 for the chapters and items on the CMR reporting form) were not mandatory to fill out by healthcare providers. For further analysis one researcher (KC) analysed all relevant incidents first. Each incident was classified using six main categories: person discovering the incident; phase of the medication process in which the incident occurred; immediate cause of the incident; nature of incident from the healthcare provider's perspective, nature of incident from the patient's perspective and the consequent harm to the patient resulting from the incident.

The category ‘person discovering the incident’ was deduced from the description of the incidents as far as possible. The subcategory ‘unknown’ was used when the report did not contain enough information to establish this characteristic.

The phase of the medication process in which the incident could have occurred was subcategorized into 9 phases: prescribing; entering the prescription into the pharmacy information system including using clinical decision support (e.g. managing drug-drug interactions, drug-disease interactions, etc.); compounding; logistics / storage of the medicines; filling ADD bag; adjustment of ADD bag; dispensing; patient monitoring and administration. These phases were derived from the classification which was used by the CMR to classify the specific phases of the medication process in which the medication incident had occurred. [21] We added new phases which were related to ADD, namely filling ADD bag and adjustment of ADD bags. Entering the prescription into the pharmacy information system was divided into three additional sub phases: entering prescription into pharmacy information system and applying clinical decision support, processing the ADD module of the pharmacy information system, and sending the ADD file to the ADD supplier. During the processing of the ADD module the pharmacy team fills in the number of medicines and times of intake.

Immediate cause is defined as a circumstance, action or influence that has triggered the incident. For the category ‘immediate cause’ no predefined subcategories were used. The researchers used the description of the incident to classify the immediate causes.

The nature of the incident was described both from the healthcare provider's perspective and from the of patient's perspective. Again, the nature of the incident (from either perspective) was classified using the description of the incident. The nature of the incident from the healthcare provider's perspective was defined as the actual error committed by the healthcare provider (e.g. entering wrong prescription). The nature of the incident from the patient's perspective was defined as the actual dispensing error (e.g. too many tablets within one ADD bag).

**Figure 2:**
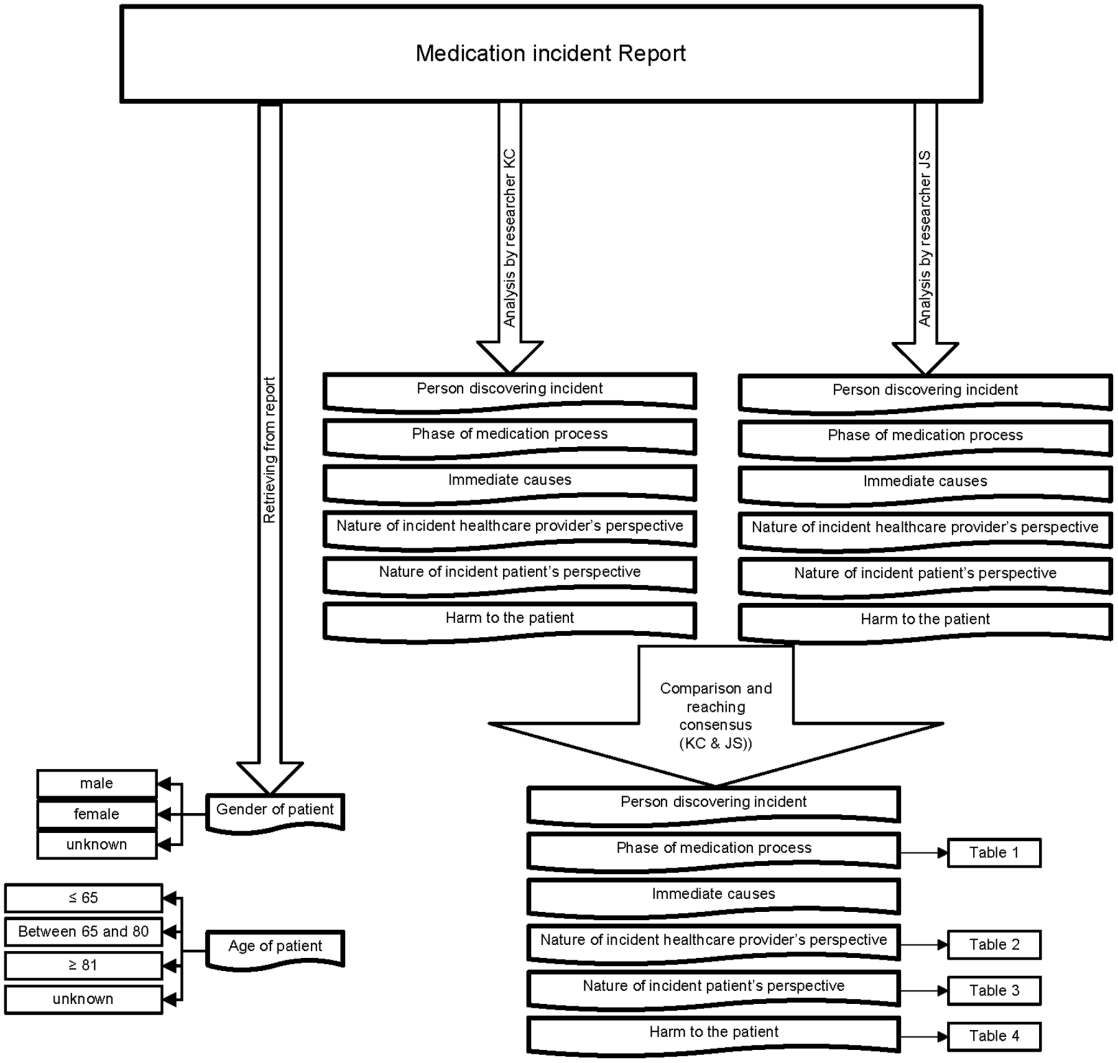
Flowchart of analysis medication incident reports.

A second researcher (JS) classified all incidents independently. Both researchers were pharmacists and participated in the CMR team for screening incidents. Figure 2 summarised the analysis of the medication incident report in a flowchart. The percentages of initial agreement between the two observers concerning the different aspects of the incidents from community pharmacies ranged from 44.1% to 61.2% and for hospital-based incidents from 29.3% to 63.4%. They subsequently came together to compare their results and to reach consensus about the incidents, which was possible for all incidents. For both the community pharmacies and the hospitals the percentages of initial agreement between the researchers were lowest (44.1% and 29.3% respectively) concerning the phase of the medication process in which the incident had occurred. In the community pharmacy-based incidents the highest percentage of agreement was for the nature of the incident from the healthcare provider's perspective. In the hospital-based incidents the highest percentage of agreement was for the person discovering the incident.

## Results

In the study period the CMR received 15,113 incidents. Healthcare providers working in community pharmacies submitted 3,685 (24.4%) incidents and those in hospitals submitted 11,428 (75.6%) incidents. In total 268 (1.8%) incidents were related to ADD: 227 (227/3685 = 6.2%) incidents from community pharmacies and 41 (41/11428 = 0.4%) incidents from hospitals. In almost half (48.9%, n = 111) of the incidents in the community pharmacies a female patient was involved and in the hospitals 26.8% (n = 11) of the incidents a female patient was involved. In the incidents derived from the community pharmacies 71 (31.3%) patients were 81 year and older, 64 (28.2%) patients were 64 year and younger and 48 (21.1%) patients had an age between 65 and 80 year. Only for three hospital incidents the age could be calculated: two (4.9%) patients had an age between 65 and 80 year, one (2.4%) patient was 81 year and older. For 41 community pharmacy incidents (18.1%) the gender of the patient was not filled in and in another 44 (19.4%) incidents the healthcare provider did not fill in the year of birth. In the hospitals the healthcare care providers did not fill in the gender and birth year for respectively 22 incidents (53.7%) and 38 incidents (92.7%).

### Person discovering the incident

Of the incidents reported by community pharmacies 23.8% (n = 54) were discovered by the patients or their family members. Other incidents were discovered by the pharmacists (12.3%, n = 28) and home care workers (7.0%, n = 16). In one incident both the pharmacist and the home care worker discovered the incident. From 119 (52.4%) incidents reported by community pharmacies the researchers could not deduce who discovered the incident.

For 73.2% (n = 30) of the hospital-based incidents, it was not clear who discovered the incident. In 4 (9.8%) incidents the nurses discovered the mistake in the ADD bag. Only in three (7.3%) incidents the patient had discovered the incident.

### Phase of the medication process

Table 1 shows the number of incidents occurring in the different phases of the medication process. No incidents occurred in the compounding, patient monitoring, and logistics / storage phases of the medication process. Many (43.6%, n = 99) incidents reported by community pharmacies occurred in the phase of entering the prescription into the pharmacy information system.

**Table 1:** Distribution of incidents occurring in the phases of medication process.

Phase of medication process	Community pharmacies n (%) N = 227	Hospitals n (%) N = 41
Prescribing	4 (2)	3 (7)
Entering into pharmacy information system		
- entering into system and applying clinical decision support	47 (20.7)	7 (17.1)
- processing ADD system*	49 (21.6)	3 (7)
- sending ADD file to ADD supplier*	3 (1)	-
Filling of ADD bag*	43 (18.9)	11 (26.8)
Adjustment of ADD bag*	19 (8.4)	-
Dispensing	23 (10.1)	1 (2)
Administration	4 (2)	4 (10)
Unknown	35 (15.4)	12 (29.3)

*additional phase of medication process for incidents concerning ADD.

### Immediate cause

In community pharmacy-based incidents a frequent immediate cause was an alteration of the medication regimen (24.2%, n = 55). Examples of alterations were the addition of a new medicine to, a change in the strength, dosage, or administration time of a medicine and stopping the use of a medicine. For 16 incidents (7.0%) a switch to another brand or generic label caused the incident. In 10 incidents (4.4%) a discharge from or admission to hospital or nursing home led to an incident. For 116 (51.1%) incidents the researchers could not deduce the immediate causes from the reports.

In hospital-based incidents, the admission to hospital, discharge from hospital and transfer to another ward were immediate causes for 15 incidents (36.6%). For 3 incidents (7.3%) the switching to another brand or generic label contributed to the incident. Alteration of the medicine regimen was the immediate cause for one incident (2.4%). For 22 (53.7%) incidents the immediate causes remained unknown.

### Nature of incident from the healthcare provider's perspective

Table 2 shows the different natures of incident from the perspective of the healthcare provider. In 76 (33.5%) community pharmacy-based incidents and 21 (51.2%) hospital-based incidents, the researchers could not deduce any nature from the description of the incident.

**Table 2:** Nature of incident from the healthcare provider's perspective.

Nature of incident	Community pharmacies n (%) N = 227	Hospitals n (%) N = 41
Fail to retrieve information about the patient	8 (3.5)	3 (7)
Selecting wrong patient	14 (6.2)	-
Choosing wrong medicine:		
- erroneous exchange	3 (1)	-
- strength	5 (2)	1 (2)
- formulation	3 (1)	1 (2)
Choosing wrong dose / frequency	13 (5.7)	2 (5)
Choosing wrong administration time	8 (3.5)	-
Choosing wrong start / end date	10 (4.4)	-
Choosing wrong duration / quantity	1 (0)	-
Entering medicine twice	1 (0)	1 (2)
Entering wrong information on administration list	5 (2)	-
Prescription was/is not processed	9 (4.0)	2 (5)
No or wrong file sent to ADD supplier	3 (1)	-
Wrong processing order in system	19 (8.4)	2 (5)
Wrong response to alert	2 (1)	-
Wrong counselling	5 (2)	-
Forgot to take out tablet of ADD bag	10 (4.4)	-
Forgot to put tablet into ADD bag	3 (1)	-
Forgot to stop order in system	9 (4.0)	1 (2)
Wrong tablet taken out of ADD bag	3 (1)	-
No or wrong cut in ADD roll	4 (2)	2 (5)
Medicine is not dispensed	2 (1)	-
Did not send stop message to pharmacy	-	1 (2)
Other:	11 (4.8)	4 (10)
Unknown	76 (33.5)	21 (51.2)

### Nature of incident from the patient's perspective

The different natures of the incidents from the patient's perspective as listed in Table 3. In community pharmacies 27.8% (n = 63) incidents resulted in too few tablets in the ADD bag and slightly less incidents (25.6%, n = 58) resulted in too many tablets in the ADD bag. Other natures of incident were about dispensing or administering the ADD bag to the wrong patient (4.8%, n = 11) and wrong information on the administration list (4.8%, n = 11). Another nature was not taking into account that the patient was using ADD bags and the patient received medicines outside the ADD bag that should have been included in the bag (5.3%, n = 12). In hospitals, many incidents involved too few or too many tablets in the ADD bag, 39.0% (n = 16) and 24.4% (n = 10).), respectively.

**Table 3:** Nature of incident from the patient's perspective.

Natures of incident	Community pharmacies n (%) N = 227	Hospitals n (%) N = 41
Too many tablets in ADD bag&	58 (25.6)	10 (24.4)
Too few tablets in ADD bag&	63 (27.8)	16 (39.1)
Wrong tablet in ADD bag&	20 (8.8)	4 (9.7)
Tablet was broken in ADD bag&	3 (1)	1 (2)
Tablet in wrong time ADD bag	5 (2)	-
No ADD roll& for patient	9 (4.0)	1 (2)
Extra ADD roll& for patient	7 (3.1)	-
Wrong information on ADD bag&	4 (2)	-
No or wrong cut in the ADD roll&	1 (0)	-
Wrong patient	11 (4.8)	1 (2)
Wrong information on administration list	11 (4.8)	-
Providing separate medicine beside the ADD bag&	12 (5.3)	2 (5)
Not providing separate medicine beside the ADD bag&	3 (1)	-
Delivering problems	3 (1)	-
Patient used separate medicine beside the ADD bag&	2 (1)	-
Did not use the medicine on the right time	-	1 (2)
Other	3 (1)	3 (7)
Unknown	12 (5.3)	2 (5)

& In an ADD bag all medicines intended for one dosing moment are gathered in disposable bags and labelled with patient data, medicine contents and the date and time for intake. Not all medication can be dispensed by the distribution robot, because specific dosage forms (e.g., suppositories, oral liquid formulations) cannot be dispensed with this system. In an ADD roll the bags with medicine (e.g. tablets) for one or two weeks are attached to each other.

### Harm to the patient

Table 4 shows the harm to the patient according to the healthcare provider who reported the incident to the CMR reporting system. In three community pharmacy-based incidents the healthcare providers reported serious temporary harm to the patient: one patient was admitted to hospital, another patient was feeling groggy and could not stand anymore, and in the third incident there was merely an indication of dizziness. The hospital-based incident with temporary serious harm did not contain enough information to deduce the type of harm to the patient.

**Table 4:** Harm to the patient.

Harm to the patient	Community pharmacy n (%) N = 227	Hospital n (%) N = 41
Incident did not reach the patient	88 (38.8)	26 (63.4)
No discomfort	98 (43.2)	7 (17.1)
Minimal/mild harm	34 (15.0)	2 (5)
Serious temporary harm	3 (1)	1 (2)
Serious permanent harm	-	-
Death	-	-
Unknown	4 (1)	5 (12)

## Discussion

As far as the authors know this is the first comprehensive study with descriptive data on the nature and consequences of medication incidents related to ADD reported by healthcare providers in community pharmacies and hospitals. A low proportion of reported medication incidents was related to ADD and especially in reported medication incidents from hospitals. Adopting the ADD in the pharmacy has introduced four new (sub) phases within the medication process. Despite the overall low number of incident reports we believe that this study adds valuable information on ADD in the pharmacy. We found that most incidents were concentrated in two typical pharmacy phases: entering the prescription into the pharmacy information system and filling the ADD bag. From our analysis we have an indication that the immediate cause of an incident was often a change in the patient's medicine regimen or relocation. The changes in the patient's medicine regimen contributed to incidents occurring in the phase of adjusting the content of the ADD bag. Such adjustments were time consuming and had to be done manually and under pressure of time by the pharmacy team.

Sinnemaki et al performed a systematic review on the outcomes of ADD: appropriateness of medication use, medication safety and costs in primary healthcare. [9] The conclusion was that controlled studies about the outcomes of the ADD bags are rare and that evidence for ADD's influence on appropriateness and safety of medication use is limited. Van den Bemt et al looked at the incidents in the administration phase and Palttala et al investigated the filling of ADD bags by ADD robots. [8], [17] The latter group observed product-dependent tablet defects during the phase of filling ADD bags. Tablet defects (tablet entirely or partially crushed, sliced, eroded, or divided into two parts) occurred in 0.15‰ to 0.20‰ of dispensed medicines. [17] In our study we found comparable incidents with broken tablets. Palttala et al also discovered unintended migration of the medicinal product to the wrong ADD bag (e.g. tablet into the afternoon ADD bag instead of the morning ADD bag). This may be comparable to our findings of too many tablets, too few tablets and wrong tablets in the ADD bag. Palttale et al found that unintended tablet migrations depended on the ADD machine used. [17]

Van den Bemt et al observed 428 incidents such as wrong administration techniques, wrong time errors, and omission errors, while we only identified eight incidents in the administration phase. [8] Such errors should be compared, however, to the administration errors that occur without the use of ADD. A study in the nephrology pediatric unit of a French hospital compared administration error rates related to ADD (plus computerized prescribing) with those occurring in the ordinary ward stock distribution system (plus handwritten prescribing). The former administration error rate was significantly lower than the latter: 22.5% (888 of 3943) versus 29.3% (189 of 646). [23] Furthermore, underreporting may play an important role: Van den Bemt et al used disguised observation to discover incidents which is known to result in much higher error frequencies.

Larsen et al investigated the effects of the use of ADD on the users' handling and consumption of medication with qualitative interviews. They discovered that for 7 of the 9 interviewed patients' excess medication was not removed from users' homes after the introduction of the ADD. [11] In our study two incidents concerned the use of separate medicines besides the use of ADD. Both patients had a stock pile of medicines and did not know that their medicines were already contained in the ADD.

### Strengths and limitations

A main strength of this study is the large number of incidents from both community pharmacies and hospitals reported to the CMR. A second strength is the independent descriptive analysis of incidents by two researchers who were both pharmacists with hands-on experience in the analysis of CMR incidents. In addition the comparison between the different healthcare settings is a plus.

A limitation is that the incidents came from a voluntary reporting system, implying that healthcare providers may have primarily focused on incidents that they considered extraordinary or especially important. And underreporting may also be present. [24] The absolute number of incidents with respect to ADD was relatively low, especially within hospitals. An issue within underreporting is selective reporting. Serious medication errors may be reported quicker and this may lead to over presentation of some types of medication incidents.

A second limitation is that not all the incidents were described in sufficient detail and that some of them hardly contained enough information for analysis. For that reason we could not perform in depth analysis for each incident to classify all the six main categories and some categories remained unknown. The quality of the reports can be enhanced by educating healthcare providers about reporting or the CMR organisation can offer a manual about good reporting practice. A third limitation was that healthcare providers could only report their incident once and could not supplement the reported incident with extra information after reporting. It is possible that healthcare providers report the incidents just after discovery and that not all the information about the incident (e.g. final harm to the patient, underlying causes) is available at that moment. This can explain why some reports hardly contain enough information. To minimise the risk that the researcher would infer details of the incident that were not actually reported, the two researchers analysed the incidents independently and met afterwards to reach consensus.

### Implications for research

This study provides a descriptive insight into the nature of the incidents associated with ADD. Future research should also focus on observations and inspections of the ADD bags. This kind of research will give insight into the absolute numbers of errors and may provide insight into specific risk factors determining errors. Furthermore, the reporting of incidents should also be done by patients, general practitioners and home care nurses to get deeper insight into ADD related incidents in all phases of the medication process. The current incidents were reported by community pharmacists and in the hospitals by nurses, physicians and hospital pharmacists. Home care nurses may have a better overview of the use and administration of ADD bag in patients' home situations. Comparing the number of ADD related incidents with the total number of ADD prescriptions could provide additional insight into the risk of using ADD. Therefore, research to retrieve the number of ADD prescriptions is necessary, although the actual risk can never be determined from reported incidents due to underreporting. In the current identification method the researcher used the flagging by the CMR team that marked all ADD incidents during the weekly screening. This identification method has not been validated and the CMR organisation needs a standard method to identify relevant incidents. Research into identification methods is necessary.

Finally, more research is needed to study the impact of ADD on elderly people, as was done in two Dutch studies. [6], [7]

### Implications for practice

ADD has implications for the workflow of the pharmacy and these new operations also need to be accompanied with prospective risk analysis and with health technology assessment (HTA). The absolute percentage of incidents related to ADD may seem low, but the use of ADD will increase further and it is necessary to pay attention to this new type of incidents in healthcare. In the implementation of ADD, healthcare providers may have focused on the advantages, but new technologies can also have unintended consequences. This descriptive study will help healthcare providers to become more aware of the most vulnerable aspects of ADD so that they can take targeted measures to reduce their unintended consequences.

To reduce the reoccurrence of ADD incidents it should be considered to perform double checks on the entering of the prescriptions and orders into the pharmacy information system, postpone alteration of patients' medication regimen when possible, avoid manual adjustments of ADD bags, follow training in the processing of ADD and to report ADD incidents adequately.

## Conclusions

ADD is just being introduced in some countries and this technology will be used more and more. Therefore it is of paramount importance that healthcare providers are aware of this kind of incidents to optimize ADD in practice. This is the first study providing descriptive data about medication incidents related to ADD in community pharmacy and hospital settings. The incidents were concentrated in two phases of the medication process: entering into the pharmacy information system and filling the ADD bags. An important recommendation for preventing reoccurrence of ADD related incidents is to perform a double check on data entering into the pharmacy information system. Furthermore extra care should be taken during and after relocation of the patient.

## Supporting Information

Appendix S1 **Chapters and items on the CMR reporting form.**
(DOC)
