# Supplementary material for: Medication Incidents Related to Automated Dose Dispensing in Community Pharmacies and Hospitals - A Reporting System Study
Source: PLoS One. 2014 Jul 24;9(7):e101686. doi: 10.1371/journal.pone.0101686 (PMC4109935; doi:10.1371/journal.pone.0101686)
Supplement: Appendix S1 — Chapters and items on the CMR reporting form. (DOC) [file pone.0101686.s001.doc]

**Appendix A: Chapters and items on the CMR reporting form**

|  | Items | Multiple choices and remarks |
| --- | --- | --- |
| **Administrative information** | | |
|  | Identification number of the healthcare practice | - |
|  | Date of reporting | - |
|  | Date on which the medication event occurred | - |
| **Data of patient** | | |
|  | Year of birth of the patient | - |
|  | Sex of the patient | - Male - Female |
| **Information about the medication event** | | |
|  | Please describe what happened | Open ended question |
|  | Which medication was involved? | - |
|  | What was the error type | - Prescribing error - Transcription error - Assembling the prescription and medication surveillance error - Compounding error - Dispensing error - Administration error - Patient monitoring error - Storage and logistic error |
|  | Did the medication event take place during a transfer of the patient (shared care)? | - Yes, during admission to hospital - Yes, during discharge of hospital - Yes, between the wards in one hospital - Yes, during out-of-hours services in the primary care - Yes, with the intensive care for thrombotic patients - Yes, namely: - No |
|  | What are the causes of the medication event? | - Technical - Organisation - Behaviour - Communication - Patient |
|  | Who makes the first error in the medication event? | List of healthcare providers. There are three different lists for the hospitals, community pharmacies and mental healthcare. |
|  | Which ward is this person involved? | List of wards in a hospital. This question exists only in the form for hospitals. |
|  | Did the medication event reach the patient? | - Yes - No |
|  | What is the harm of the medication event to the patient? | - No discomfort - Minimal/mild harm - Seriously temporary harm - Seriously permanent harm - Death - Unknown |
|  | What could be the potential harm to the patient? | - Scale from 1 to 5 or unable to estimate |
| **Questions to notify an alert** | | |
|  | How much is the risk of recurrence? | - Unlikely, less than 1 times a year - Rare, less than 5 times a year - Possible within a few months - Probably within a few days - Almost sure within a few hours/days - Unable to estimate |
|  | Can other healthcare providers learn from this reported medication event? | - Scale from 1 to 5of unable to estimate |
|  | Is this reported medication event suitable for an alert? | - Yes, this is an alert, CMR organisation will contact the informant for detailed information. - No, this is not an alert. - Please let the CMR organisation contact the informant. |
